# Pre-Vascularized 3-Dimensional Skin Substitutes Promote Angiogenesis and Tissue Repair in a Murine Model of Refractory Skin Ulcers

**DOI:** 10.3390/jfb16110409

**Published:** 2025-11-03

**Authors:** Shota Tojo, Hiromi Miyazaki, Takami Saiki, Yasuyuki Tsunoi, Shingo Nakamura, Ryuichi Azuma

**Affiliations:** 1 Department of Plastic and Reconstructive Surgery, National Defense Medical College, Saitama 359-8513, Japan; shota1005@ndmc.ac.jp (S.T.); azuma@ndmc.ac.jp (R.A.); 2 Division of Biomedical Engineering, National Defense Medical College Research Institute, Saitama 359-8513, Japan; res372@ndmc.ac.jp; 3 Division of Bioinformation and Therapeutic Systems, National Defense Medical College Research Institute, Saitama 359-8513, Japan; ytsunoi@ndmc.ac.jp

**Keywords:** angiogenesis, regional blood flow, skin substitutes, skin ulcer, wound healing

## Abstract

Restoring blood flow is crucial for treating refractory ulcers. Despite advancements in various biomaterials, none incorporating pre-formed blood vessels have been commercialized. To address this, we developed a pre-vascularized three-dimensional (3D) skin substitute (PV-3D skin) designed to enhance healing when treating refractory ulcers. This study aimed to evaluate the therapeutic role of PV-3D skin transplantation in refractory ulcer models, induced by applying mitomycin C to wounds in severe immunodeficient mice. The wounds were then treated with PV-3D skin, non-vascularized 3D skin, skin grafts, or wound dressings. The PV-3D skin group demonstrated healing dynamics comparable to those of the skin graft group, with similar tissue morphology and wound temperature changes. Furthermore, at day 7 post-transplantation, the PV-3D skin group demonstrated significantly higher hypoxia-inducible factor 1-alpha expression levels compared to the 3D skin group. By day 14, the PV-3D skin group exhibited a significantly larger vascular area compared to the 3D skin group. Notably, PV-3D skin treatment stimulated host-derived angiogenesis, thereby enhancing wound healing and reducing the recurrence of refractory ulcers. These results suggest that PV-3D skin transplantation offers a promising therapeutic approach for refractory ulcers, especially in terms of angiogenesis.

## 1. Introduction

Chronic wounds, including refractory ulcers, pose significant challenges in clinical management, as standard therapies have shown limited efficacy. Conventional treatment typically involves debridement to remove necrotic tissue, followed by non-surgical approaches to promote granulation tissue formation [1,2]. Once sufficient granulation tissue develops, epithelialization usually progresses, leading to wound healing. However, skin grafting or flap procedures may be required in cases of extensive ulcers or when granulation tissue remains immature and non-epithelializing. Conservative treatment approaches include the application of growth factors such as basic fibroblast growth factor (FGF), prostaglandins (PGs), and cyclic adenosine monophosphate (cAMP) derivatives, along with wound dressings like hydrocolloids, polyurethane foams, and negative-pressure wound therapy. Biomaterials such as dermal–epidermal composite substitutes, dried amniotic membranes, and collagen-based extracellular matrices (e.g., small intestinal submucosa) are highly effective for refractory ulcers [3,4,5]. Despite these therapeutic advancements, chronic wounds often remain challenging to heal due to underlying conditions such as venous stasis, atherosclerosis, radiation-induced tissue damage, prolonged corticosteroid use, or diabetes, all of which impair blood flow and fibroblast function. These factors hinder granulation tissue formation and delay wound healing [6,7]. Thus, optimizing tissue perfusion is crucial for successful treatment [8,9], highlighting the urgent need for advanced biomaterials and regenerative strategies to address these underlying pathophysiological challenges.

We have developed a pre-vascularized three-dimensional (PV-3D) skin substitute with a two-layer structure (epidermal and vascularized dermal layers) [10]. This design aims to overcome the challenge of tissue repair failure caused by insufficient blood flow, a critical factor in the impaired healing of chronic refractory wounds. Notably, no previous studies have reported using vascularized, three-dimensional cultured skin composed entirely of human skin-derived cells without relying on scaffolds such as Matrigel to treat refractory ulcers. If successful, this approach could be a novel and effective therapeutic option for refractory ulcers.

This study aimed to evaluate the therapeutic efficacy of PV-3D skin transplantation in a refractory ulcer mouse model.

## 2. Materials and Methods

### 2.1. Cell Culture

Neonatal normal human dermal fibroblasts (NHDFs, CC-2509; Lonza, Basel, Switzerland) were cultured in Dulbecco’s modified Eagle’s medium (DMEM; Nacalai Tesque, Kyoto, Japan) supplemented with 5% fetal bovine serum (Gibco, Waltham, MA, USA) and 1% antibiotic-antimycotic mixed stock solution (Nacalai Tesque), and were then used until passage 6. Human dermal microvascular endothelial cells (HMVECs, CC-2813; Lonza) were cultured in endothelial growth medium (EGM-2MV, Lonza) and used at passage 5. Neonatal human epidermal keratinocytes (NHEKs, KK-4009; Lifeline Cell Technology, Frederick, TX, USA) were maintained in HuMedia–KG2 medium (KURABO, Osaka, Japan) and used at passage 3. Cells were passaged upon reaching 80–90% confluency using 0.25% trypsin/ethylenediaminetetraacetic acid solution (Thermo Fisher Scientific Inc., Waltham, MA, USA). The culture media were changed every 1–2 days.

### 2.2. Construction of Vascularized 3D Skin Substitute

In a previous study [10], a 3D skin substitute incorporating a vascular network was constructed using a cell accumulation approach combined with a layer–by–layer cell coating technique [11]. Briefly, NHDFs were alternately suspended with 0.04 mg/mL fibronectin (FN; Sigma-Aldrich, St. Louis, MO, USA) or 0.04 mg/mL gelatin (G; Fujifilm Wako, Osaka, Japan) and washed with phosphate-buffered saline (PBS) between each coating step. After four repetitions of this cycle, cells were again suspended with FN solution and washed with Dulbecco’s PBS. To construct a multilayer tissue containing blood vessel network, the coated NHDFs (1 × 10^7^ cells) were cultured alone or co-cultured with HMVECs (2 × 10^5^ cells) in 12-well insert with a 0.4 μm pore size (Corning, Corning, NY, USA) for 24 h. NHEKs (3 × 10^5^ cells) in a 1:1 *v*/*v* ratio of DMEM and HuMedia–KG2 were subsequently seeded on the dermal layer coated with 0.04 mg/mL collagen type IV solution (from human placenta, Sigma-Aldrich), submerged in the mixed medium and cultured for 24 h. Epidermal differentiation was induced by replacing the mixed medium with 25 μg/mL L-ascorbic acid (Fujifilm Wako), followed by air-liquid interface cultivation for 5 days. Prior to grafting, a subset of the PV-3D skin constructs was reserved for quality control; pre-vascularization was verified by immunostaining for endothelial markers (e.g., human CD31) to confirm the presence of interconnected capillary-like networks. Representative images are provided in Figure S1B.

### 2.3. Animal Experiments

**Figure 1:**
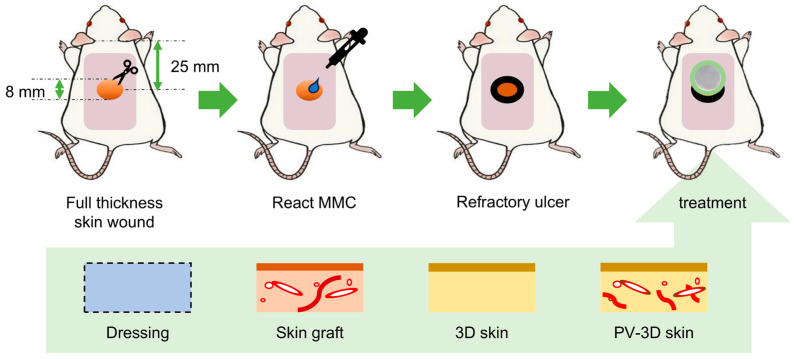
Schematic illustration of the treatment of refractory skin ulcers in mice with severe combined immunodeficiency. MMC: mitomycin C; PV-3D: pre-vascularized three-dimensional.

Following all ethical regulations, animal experiments in this study received approval from the Animal Ethics Committee of the National Defense Medical College (Approval No. 22062: Study on the Treatment of Refractory Ulcers Using 3D Cultured Skin, 14 March 2023). For application of human derived skin substitutes in transplantation, male mice with severe combined immunodeficiency (SCID), aged 8–9 weeks (25–30 g), were obtained from Charles River Laboratories (Kanagawa, Japan). After induction of inhalation anesthesia with isoflurane, the dorsal hair was shaved, and depilatory cream (Epilat^®^; Kracie Home Products, Tokyo, Japan) was applied. A full-thickness wound (8 mm in diameter) was created at the center of the back and excised skin was used as graft. To create a refractory wound, mitomycin C (MMC; 20 μg in 20 μL of a solution containing 10% (*v*/*v*) ethanol and 90% (*v*/*v*) ethylene glycol; Fujifilm Wako) was applied to the wound for 10 min [12]. After washing the wound with saline, mice were divided into four groups and treated with either PV-3D skin, 3D skin substitutes (without vasculature), syngeneic skin grafts, or wound dressing. The wound was protected with a silicone-faced dressing (SI–Aid^®^; ALCARE Co., Tokyo, Japan) and secured with transparent film dressing (Opsite^®^; Smith & Nephew, Tokyo, Japan) (Figure 1). The wound dressing comprised an inert, non-adherent silicone mesh without any bioactive components such as antimicrobials, drugs, biologics, or growth factors. This dressing was used solely as a neutral contact layer to prevent adhesion and to allow exudate to pass to a secondary absorbent pad. A circular silicone ring was secured to prevent wound contraction during long-term observation of 28 days. At 7, 14, and 28 days post-transplantation, the mice were euthanized; the dorsal skin, including the graft, was then harvested for histological evaluation. During tissue harvesting, each wound was inverted to expose the underside, and was photographed at a fixed distance under standardized conditions.

### 2.4. Evaluation of Wound State

On postoperative days 3, 7, 10, and 14, the surface temperature surrounding the wounds was assessed using an infrared thermography non-contact thermometer (FLIR ONE^®^ GEN 3; Teledyne FLIR LLC, Wilsonville, OR, USA), and blood flow was measured by laser speckle blood flow imaging system (OMEGAZONE OZ-2; Omegawave Inc., Osaka, Japan).

### 2.5. Histological Observation and Immunohistochemistry

Tissues were fixed in 10% neutral buffered formalin for 24 h and embedded in paraffin. Hematoxylin and Eosin (H&E) and Trichrome (Abcam, Cambridge, UK) staining were performed according to the manufacturer’s recommendations.

For immunohistochemical analysis, sections were stained using primary antibodies after blocking with normal goat serum. Primary antibodies against human leukocyte antigen (HLA)-Class I ABC (1:2500, ab70328, clone EMR8-5; Abcam), human and mouse-specific Fibroblast activation protein (FAP; 1:200, ABIN2929221, clone AA 542-761; antibodies.com, Atlanta, GA, USA), human-specific FAP (1:200, ab314456, clone RM1080; Abcam), human-specific CD31 (1:200, NBP2-15202, clone C31.3; Novus Biologicals, Centennial, CO, USA), mouse-specific CD31 (1:100, DIA-310, clone sz31; dianova, Hamburg, Germany), and hypoxia-inducible factor 1-alpha (HIF-1α; 1:400, ab228649, clone C-terminal; Abcam), Ly6g/6c (1:500, ab25377, clone RB6-8C5; abcam) were used. Before immunostaining with mouse-derived antibodies, we treated the sections with endogenous mouse immunoglobulin G (IgG) blocking reagent (M.O.M.^®^ Immunodetection Kit; Vector Laboratories, Burlingame, CA, USA). The sections were reacted with anti-mouse or anti-rabbit IgG ImmPRESS-HRP reagent (Vector Laboratories) and visualized using DAB (Dako, Glostrup, Denmark). The stained tissue sections were observed under a microscope (BZ-X710; Keyence, Osaka, Japan). Wound size, thickness of the epidermal and dermal layers, and immunoreactivity of the stained areas were quantified using ImageJ software version 1.54p (National Institutes of Health, Bethesda, MD, USA).

### 2.6. Statistical Analysis

Statistical analyses were performed using GraphPad Prism version 9.5.1 (GraphPad Software, San Diego, CA, USA). Comparisons between two groups were performed using an unpaired *t*-test, whereas comparisons among multiple groups were performed using ANOVA followed by the Tukey–Kramer test. A linear mixed-effects model was used to analyze wound temperatures, with group, time, and their interaction included as fixed effects, and individual animal ID included as a random effect to account for repeated measurements. Statistical significance was defined as a *p*-value < 0.05. Data are presented as mean ± standard error.

## 3. Results

### 3.1. PV-3D Skin Accelerated Wound Healing in Refractory Ulcer

**Figure 2:**
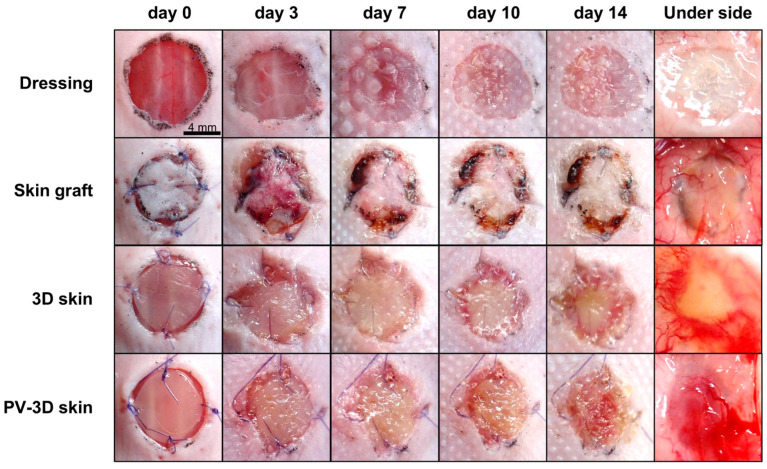
Post-transplantation macroscopic images of the wound area in the recipient mice.

In both the PV-3D and 3D skin groups, the wound surface appeared white on day 7 post-grafting. On day 14, disparities in the progression of wound healing became more discernible: the 3D skin group exhibited residual white tissue in the center, whereas the PV-3D skin group displayed signs of crust detachment, revealing underlying red tissue. Conversely, the skin graft group exhibited partial dehiscence and necrosis at the wound edges, whereas the wound dressing group was found to have a translucent wound bed and hypogranulation (Figure 2).

**Figure 3:**
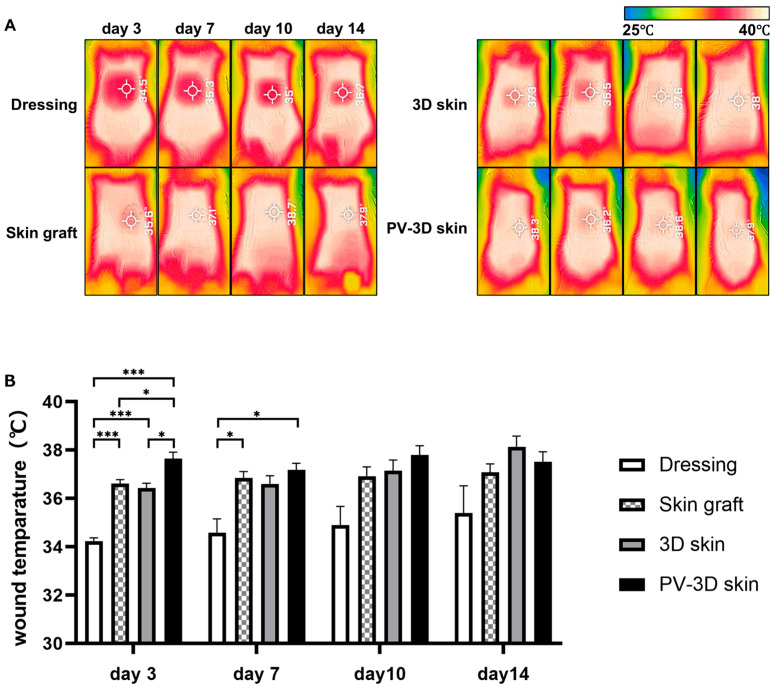
Evaluation of the therapeutic response in experimental models using infrared thermographic imaging. (**A**) Representative observation of the wound temperature. (**B**) Comparative analysis of wound temperatures across groups at each time point (*n* = 4–8 animals/time point). Data are represented as mean ± standard error of the mean. * *p* < 0.05, *** *p* < 0.001.

To further assess healing, a thermographic analysis was conducted. On day 3 after grafting, the PV-3D skin group showed significantly higher wound temperatures than the 3D skin group (*p* < 0.05), approaching normal skin temperature. The wound temperature in the wound dressing group was significantly lower than that in the other groups, suggesting a delayed thermal recovery process during the inflammatory phase of wound healing. On day 7, both the PV-3D skin and skin graft groups maintained temperatures similar to that of the surrounding skin, no significant differences observed among the groups after day 10. Importantly, throughout the observation period, no abnormal temperature increases indicative of infection were detected (Figure 3A,B).

**Figure 4:**
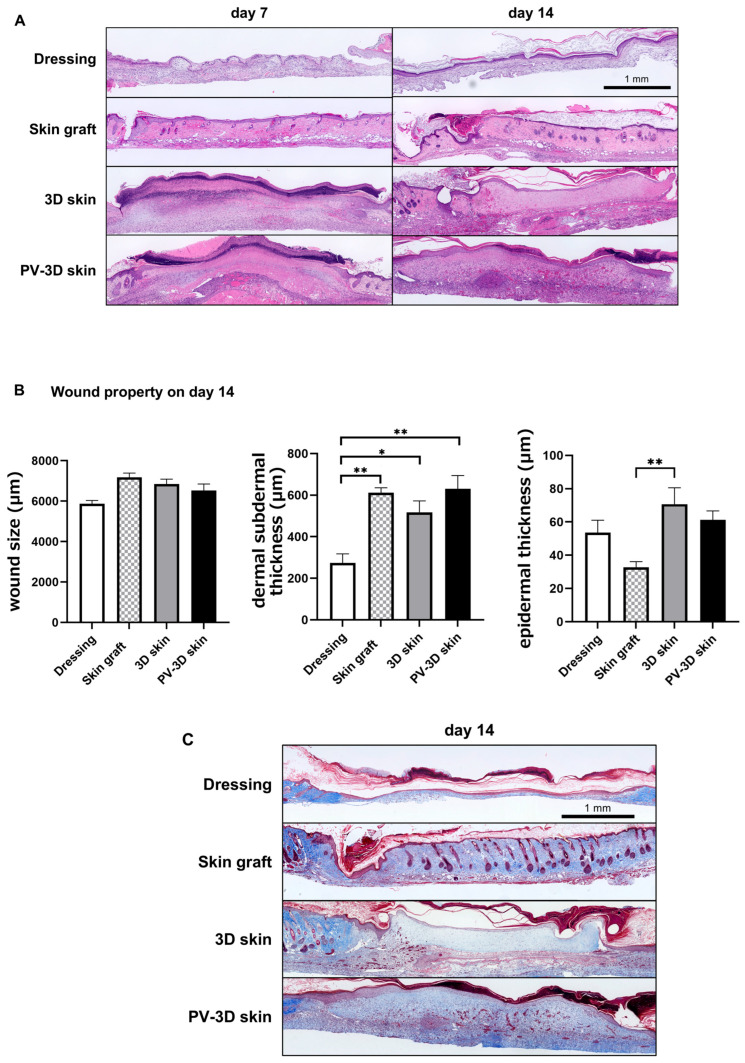
Histological analysis of the skin. (**A**) Hematoxylin and eosin staining performed on days 7 and 14 post-transplantation. (**B**) Quantitative analysis of wound diameter and the thickness of the epidermis, dermis, and subdermal layer on day 14 (*n* = 6–12 animals). Data are represented as mean ± standard error of the mean. * *p* < 0.05, ** *p* < 0.01. (**C**) Masson’s Trichrome staining on day 14.

Histological observations provided further insights into wound healing. On day 7, the PV-3D and 3D skin groups exhibited signs of engraftment and crust formation, which subsequently underwent detachment. In the skin graft group, hair follicle reduction and marginal necrosis at the wound edges were observed; however, the epidermal and dermal structures remained, suggesting a tendency toward engraftment. Conversely, the wound dressing group exhibited minimal granulation tissue without epithelialization (Figure 4A). On day 14, the dermal and subdermal tissue thickness in the wound dressing group was significantly lower compared to its thickness in other groups (*p* < 0.05); however, the wound diameter did not differ significantly (Figure 4B). A critical marker of wound remodeling, collagen deposition, was also analyzed. On day 14, the PV-3D skin group exhibited widespread collagen deposition, exhibiting a larger collagen-stained area in the Skin graft and PV-3D skin group (Figure 4C). These findings suggest that PV-3D skin enhances extracellular matrix formation, potentially improving wound stability.

**Figure 5:**
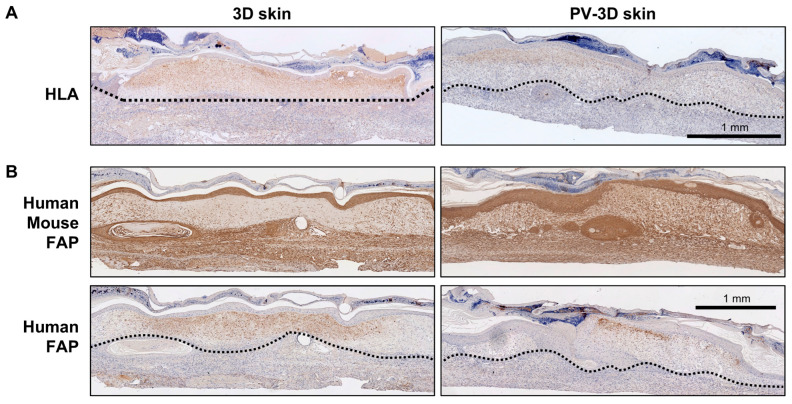
Immunohistochemical analysis of transplanted tissues on day 14. (**A**) Human leukocyte antigen and (**B**) Fibroblast activation protein staining. Dashed black lines indicate the interface between the transplanted tissue and the mouse wound bed.

To investigate the cellular composition of the grafted tissues, immunohistochemical analysis was performed. On day 14 post-grafting, HLA staining was observed in the upper wound area of 3D and PV-3D skin groups, confirming the presence of human-derived cells in the grafted regions (Figure 5A). Subsequent analysis employing FAP staining, which detects human and mouse FAP, revealed pervasive staining throughout the wound area. However, staining with an antibody specific to human FAP revealed positive signals confined to the upper wound region (Figure 5B). Notably, the PV-3D skin group exhibited fewer positive areas than the 3D skin group, suggesting potential disparities in cellular dynamics between these two skin substitutes.

### 3.2. Influence of Vascular Structure on Wound Healing

The results of the study indicate that the PV-3D skin substitute demonstrated wound healing dynamics closely aligned with those of normal skin. Given the established role of vascularization in skin graft survival and wound healing, the subsequent investigation focused on the role of vascular structures in the 3D and PV-3D skin groups.

**Figure 6:**
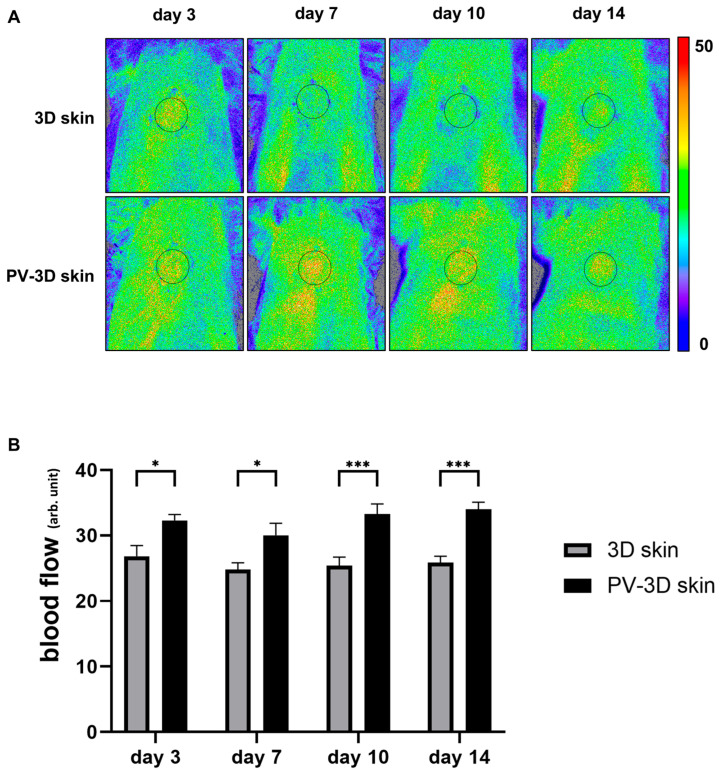
Blood flow monitoring in the pre-vascularized three-dimensional (3D) skin substitute and 3D skin groups following transplantation. (**A**) Representative laser speckle contrast images. (**B**) Quantitative analysis of blood flow changes at each time point (*n* = 6 animals/time point). Data are represented as mean ± standard error of the mean. * *p* < 0.05, *** *p* < 0.001.

Laser speckle contrast imaging revealed that beginning on day 3 post-grafting, the blood flow in the PV-3D skin group was significantly higher than in the 3D skin group (*p* < 0.05; Figure 6A,B).

**Figure 7:**
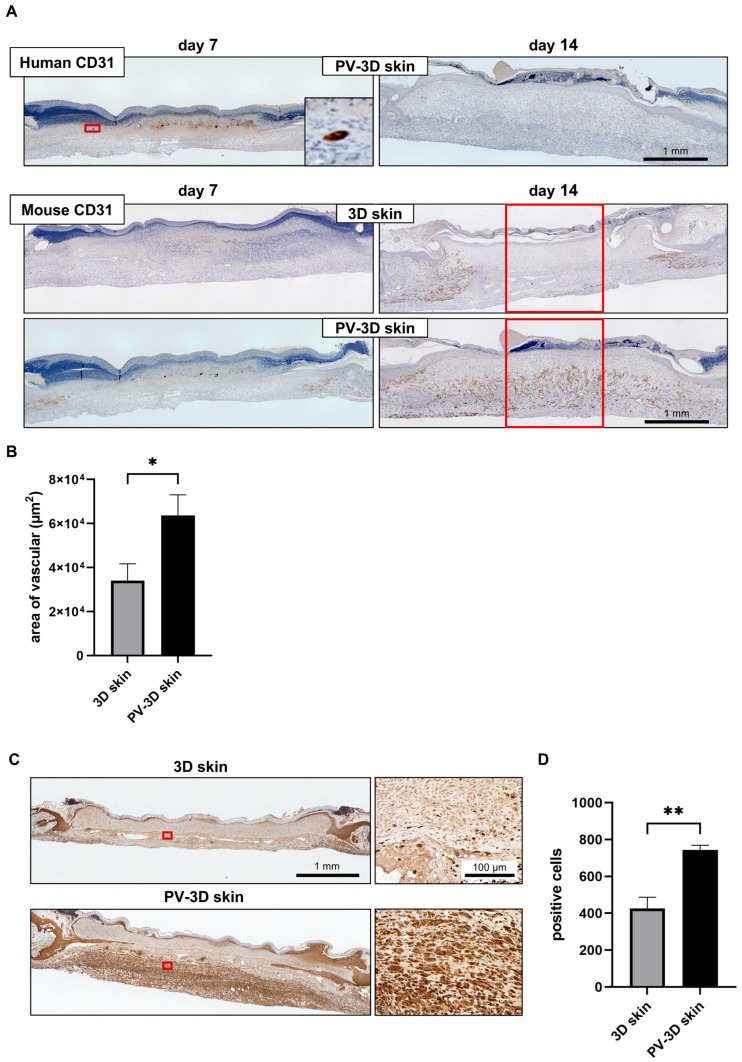
Immunohistochemical analysis at the wound site. (**A**) Human-specific CD31 and mouse-specific CD31 staining (*n* = 11–12 animals) on days 7 and 14 post-transplantation. (**B**) Quantification of the area occupied by mouse-derived blood vessels on day 14. (**C**) Hypoxia-inducible factor 1-alpha (HIF-1α) staining at the wound site on day 7. (**D**) Quantification of the HIF-1α stained cells (*n* = 5 animals). Data are represented as mean ± standard error of the mean. * *p* < 0.05, ** *p* < 0.01.

To further assess neovascularization, we performed CD31 staining, a marker of endothelial cells. On day 7, the PV-3D skin group exhibited some positive CD31 staining; however, by day 14, no human CD31-positive staining was detectable. On day 7, 3D and PV-3D skin groups exhibited host-derived CD31-positive blood vessels surrounding the grafted tissue. However, by day 14, the PV-3D skin group displayed abundant blood vessels in the wound center, whereas the 3D skin group retained blood vessels primarily around the wound periphery (Figure 7A). Quantitative analysis confirmed that the PV-3D skin group had a significantly larger blood vessel area in the wound center compared to the 3D skin group (6.4 ± 0.94 × 104 μm^2^ vs. 3.4 ± 0.76 × 104 μm^2^, *p* < 0.05; Figure 7B).

To further explore the mechanisms underlying these differences, we assessed HIF-1α expression, a key regulator of hypoxia-induced angiogenesis. On day 7 post-grafting, HIF-1α stained cells in the wound center was significantly greater in the PV-3D skin group (743 ± 25.5 cells) than in the 3D skin group (426 ± 61.2 cells, *p* < 0.05; Figure 7C,D). This finding suggests that PV-3D skin fosters a microenvironment conducive to angiogenesis, thereby further substantiating its role in promoting wound healing.

### 3.3. Long Term Observation of PV-3D Skin

**Figure 8:**
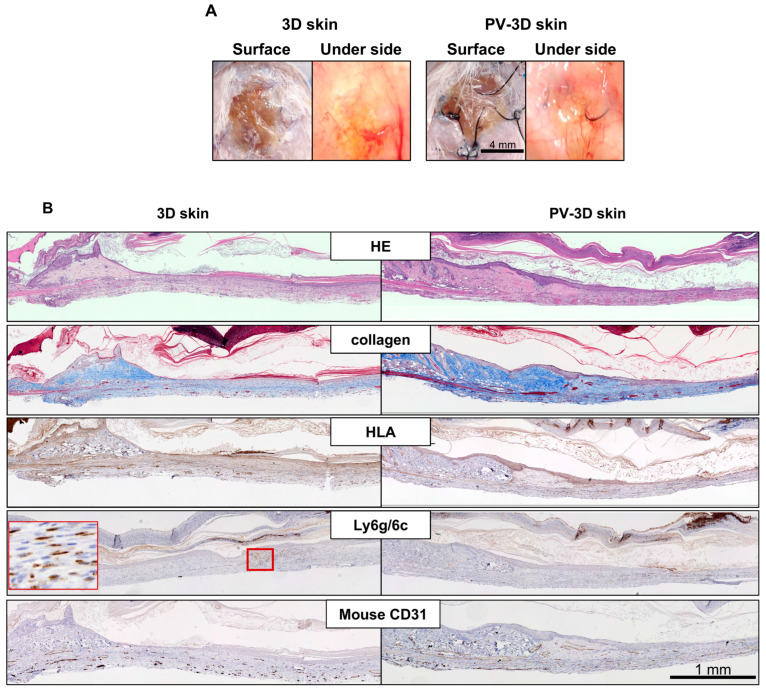
Wound site images on day 28. (**A**) Macroscopic images of the wound area and (**B**) Histological images at the wound site.

Since wound contraction exerts a significant influence on long-term assessments beyond 2 weeks, experiments were conducted with the application of a silicone ring to prevent contraction. Both 3D skin and PV-3D skin formed eschars macroscopically by 4 weeks (Figure 8A), which subsequently detached from the healed wound bed. Hematoxylin and eosin staining revealed that PV-3D skin was covered with an epidermis of comparable thickness to the surrounding murine epidermis, whereas partial epidermal loss was observed in 3D skin. Collagen staining showed weaker staining in 3D skin, while PV-3Dskin exhibited more intense staining. HLA staining was negative in both PV-3D skin and 3D skin. Immunohistochemistry for Ly6g/6c, a marker of inflammatory cells, demonstrated positive signals within the healing tissue of 3D skin, but no positivity in PV-3D skin. Murine blood vessels were observed throughout the wound area in both 3D skin and PV-3D skin (Figure 8B).

## 4. Discussion

This study demonstrates that PV-3D skin accelerates healing of mitomycin C (MMC)-induced refractory ulcers. Successful treatment of chronic wounds relies on improving local blood perfusion, as inadequate vascularization is a significant barrier to wound healing. Importantly, direct anastomosis was not observed between PV-derived and host vessels was confirmed, and human CD31^+^ areas were scarcely detected on day 14. On the other hand, by day 14 post-transplantation, sufficient host-driven vascular structures within the grafted skin-like tissue indicated enhanced blood flow and successful healing. Therefore, the role of PV-3D skin in this model can be better characterized as creating a supportive microenvironment that enables and guides host-mediated repair, rather than as a functional graft vasculature.

Thermographic analysis revealed that commencing on day 3 post-transplantation, the PV-3D skin group exhibited temperatures comparable to those of the surrounding healthy tissue, similar to conventional skin grafts. This finding suggests the presence of an epidermal barrier that reduces evaporative water loss and heat dissipation [13,14]. Furthermore, the enhanced heat retention observed in the PV-3D skin group indicates augmented local blood flow perfusion, a critical factor in wound healing. Skin temperature is a well-established indicator of vascularization, reflecting inflammation or increased metabolic activity. Recent studies employing infrared thermographic imaging have indicated that wound temperature may serve as a predictive marker for graft integration and healing. Consequently, thermographic observations may assess the engraftment of transplanted tissues [15]. The findings of our study suggest that PV-3D skin was integrated, thus indicating its favorable engraftment.

The assessment of blood flow using laser speckle contrast imaging, a well-established method for evaluating microcirculatory dynamics in skin wounds [16,17,18,19], supported the finding of increased perfusion in the wound area following PV-3D skin transplantation. Histological analysis performed on day 7 post-transplantation indicated an increase in host-derived vascular structures around the PV-3D skin, and by day 14, blood vessel formation was evident within the wound site. These findings indicate that early enhancement of blood flow in both the graft and underlying tissue improved wound healing outcomes. This observation aligns with those of the previous report highlighting the role of vascularized grafts in facilitating granulation tissue formation and neovascularization [10]. Given that blood vessels derived from cultured PV-3D skin were barely expressed in the transplanted wound after 14 days, these findings suggest that they reflected increased blood flow around and beneath the graft up to day 7, but then reflected blood flow within the graft via newly formed murine vessels after day 10.

Fibroblast activation protein (FAP) is expressed by activated fibroblasts and epithelial cells during wound healing [20]. In this study, FAP staining on day 14 revealed activation of mouse-derived epidermal cells and fibroblasts in the PV-3D skin group, suggesting enhanced epithelial-dermal interactions that are essential for effective wound healing. In the mid-layer of the wound, regions lacking HLA and human FAP staining were detected. However, based on tissue morphology, these areas were likely derived from the cultured skin. Considering these points, histology along the time course supports a host-driven mechanism. Post-engraftment, the cultured skin may have served as a scaffold for recruiting and activating host-derived fibroblasts and endothelial cells, facilitating tissue regeneration.

The MMC model recapitulates key features of refractory ulcers through inhibition of DNA synthesis and consequent impairment of fibroblast and endothelial dynamics [12,21,22,23,24,25,26,27,28]. Within this stringent context, the PV-3D skin group showed the presence of fibroblasts and vascular structures of host-derived cells and collagen production, suggesting that PV-3D skin improved the refractory ulcer condition and wound healing by enhancing dermal remodeling and vascular integration. The observed benefits are consistent with reports that biomaterials containing endothelial components can enhance blood perfusion and healing [29,30,31]. It is known that angiogenesis can occur along decellularized vascular and luminal structures [32,33]. Likewise, despite the lack of direct vascular anastomosis with host vessels, residual vascular structures and endothelial cells within the PV-3D skin likely contributed to early tissue perfusion and neovascularization at the wound site. As healing progressed, partial degeneration of the PV-3D skin may have occurred, potentially influenced by the hostile microenvironment of the MMC-induced refractory ulcer. However, by day 14, the human endothelial structures were all plausibly shed or replaced in association with the progression of host-derived neovascularization. Moreover, cultured skin with a well-organized epidermal-dermal structure alters refractory ulcers from a non-healing stage to one conductive to regeneration [34,35]. These interpretations differ from out acute full-thickness wound model, in which early restoration of flow and anastomosis between graft-derived and host vessels were observed [10]. The present MMC model imposes a more hostile microenvironment for endothelial survival and proliferation, plausibly shifting the dominant mechanism from direct graft vascular function to indirect augmentation of host neovascularization and perfusion.

Angiogenesis is crucial in wound healing [7,8], with HIF-1α playing a central role by stimulating the production of pro-angiogenic factors such as FGF, VEGF, and cytokines like TNF-α [36,37,38]. However, in refractory ulcers, this hypoxia-driven response is often impaired, leading to delayed wound healing [37]. Since HIF-1α is activated under hypoxic conditions and regulates endothelial cell function, PV-3D skin transplantation likely helped normalize the hypoxic response at the wound site, thereby promoting HIF-1α expression and facilitating angiogenesis. This mechanism could explain the increased vascularization and improved wound healing in the PV-3D skin group. Although thermography and laser Doppler flowmetry demonstrated increased blood flow up to day 7, the expression of HIF-1α was also observed at day 7, which may appear contradictory at first glance. However, in the normal wound healing process, HIF-1α expression can persist for approximately 7 days after injury [36,39], and the present findings may therefore reflect the sustained therapeutic effect on refractory ulcers.

Recurrence is a major concern in chronic refractory ulcers, often attributed to the fragility of regenerated epidermis and prolonged inflammation [6,40]. Long-term histological observations of PV-3D skin suggested a low likelihood of recurrence and minimal inflammation. This is consistent with the maintenance of supporting tissue and proper wound architecture, as indicated by collagen staining. Regarding the human tissue, both PV-3D skin and 3D skin had largely disappeared by 4 weeks, likely reflecting replacement by host murine tissue, which was already evident by 2 weeks of observation. Although SCID mice are largely immunodeficient, xenografts are not completely free from rejection. If it occurs, reported timelines for graft rejection and loss in SCID mice generally begin at least 4–6 weeks after transplantation [41,42], supporting the idea that host tissue replacement predominates during the healing process. Additionally, the absence of functional T and B cells in SCID mice alters the inflammatory course compared to normal wound healing, although innate immune responses still occur [43,44,45]. Immunohistochemistry for Ly6g/6c, a marker of macrophages and neutrophils, revealed only few inflammatory cells in PV-3D skin over the long term, suggesting that this approach may be effective in preventing chronic inflammation associated with refractory ulcers.

This study has several limitations. Firstly, although this study utilized a faithful MMC-induced ulcer model, further investigation is required to determine whether similar mechanisms apply to other non-healing models, such as ischemic, congestive/hyperemic, and diabetic ulcers [46,47,48,49]. Further work should test the use of PV-3D skin in (i) ischemic flap/venous-stasis models and (ii) diabetic models (e.g., db/db or streptozotocin-induced), by integrating longitudinal perfusion imaging with assessments of graft-host anastomosis and multiplexed profiling of the wound bed. These studies should examine stromal–immune–endothelial crosstalk, focusing on HIF-1α dependent responses and FAP positive stromal activation, to define how PV-3D skin modulates non-healing microenvironments. While these factors are beyond the scope of the present study, they represent important directions for further investigation.

Overall, this study demonstrated that PV-3D skin transplantation promotes wound healing in a drug-induced refractory ulcer model by enhancing vascularization and tissue regeneration. In particular, the PV-3D skin accelerates early perfusion and angiogenic activity, as evidenced by increased HIF-1α expression and subsequent host CD31^+^ vascular ingrowth, together with progressive matrix remodeling and tissue maturation. Given that inadequate blood perfusion is a major challenge in the treatment of chronic wounds, these findings highlight the potential of PV-3D skin as an advanced biomaterial capable of overcoming ischemia-related tissue repair failure.

## 5. Conclusions

In this study, we developed a novel biomaterial containing pre-formed blood vessels that could aid in restoring the blood flow necessary to treat refractory ulcers and evaluated its effectiveness. Although long-term validation and safety testing using large animal models are necessary next step, the PV-3D skin represents a promising therapeutic strategy for refractory ulcers, potentially contributing to the development of next-generation skin substitutes with enhanced regenerative capabilities.

## Data Availability

The raw data supporting the conclusions of this article will be made available by the corresponding authors upon request.

## Supplementary Materials

The following supporting information can be downloaded at https://www.mdpi.com/article/10.3390/jfb16110409/s1, Figure S1: Engineering of human pre-vascularized full-thickness skin substitute.

## Abbreviations

The following abbreviations are used in this manuscript:

3D	three-dimensional
cAMP	cyclic adenosine monophosphate
DMEM	Dulbecco’s modified Eagle’s medium
FAP	Fibroblast activation protein
FGF	fibroblast growth factor
FN	fibronectin
G	gelatin
H&E	Hematoxylin and Eosin
HIF-1α	hypoxia-inducible factor 1-alpha
HLA	Human leukocyte antigen
HMVECs	Human dermal microvascular endothelial cells
IgG	immunoglobulin G
MMC	mitomycin C
NHDFs	Neonatal normal human dermal fibroblasts
NHEKs	Neonatal human epidermal keratinocytes
PBS	phosphate-buffered saline
PGs	prostaglandins
PV-3D skin	pre-vascularized three-dimensional skin substitutes
SCID	severe combined immunodeficiency
