# Supplementary figures and images for: Pre-Vascularized 3-Dimensional Skin Substitutes Promote Angiogenesis and Tissue Repair in a Murine Model of Refractory Skin Ulcers
Source: J Funct Biomater. 2025 Nov 3;16(11):409. doi: 10.3390/jfb16110409 (PMC12653047; doi:10.3390/jfb16110409)

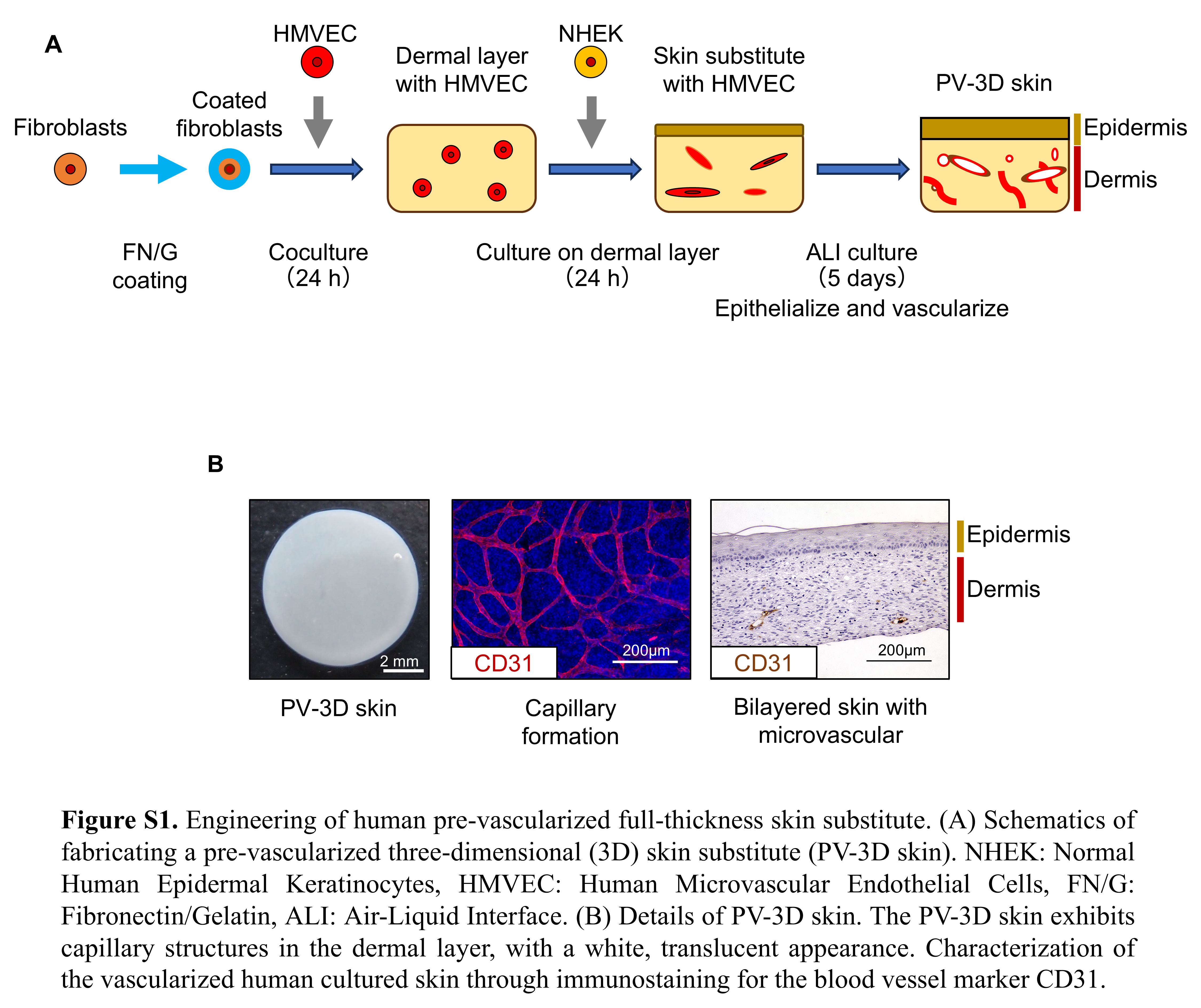

Supplement: Supplementary file 1 [file jfb-16-00409-s001.zip › jfb-3942359-supplementary.TIF]
